# Mental health integrated care models in primary care and factors that contribute to their effective implementation: a scoping review

**DOI:** 10.1186/s13033-024-00625-x

**Published:** 2024-02-09

**Authors:** Anton N. Isaacs, Eleanor K. L. Mitchell

**Affiliations:** 1 Monash University School of Rural Health, Sargeant Street, PO Box 723, Warragul, VIC 3820 Australia; 2 Monash University School of Rural Health, Corner of Victoria Street & Day Street, PO Box 1497, Bairnsdale, VIC 3875 Australia

**Keywords:** Primary health care, Integrated care, Collaborative care, Models, Organizational, Patient-Centered Care, Care coordination, Mental disorders, Mental health services

## Abstract

**Background:**

In the state of Victoria, Australia, the 111-day lockdown due to the COVID-19 pandemic exacerbated the population’s prevailing state of poor mental health. Of the 87% of Australians who visit their GP annually, 71% of health problems they discussed related to psychological issues. This review had two objectives: (1) To describe models of mental health integrated care within primary care settings that demonstrated improved mental health outcomes that were transferable to Australian settings, and (2) To outline the factors that contributed to the effective implementation of these models into routine practice.

**Methods:**

A scoping review was undertaken to synthesise the evidence in order to inform practice, policymaking, and research. Data were obtained from PubMed, CINAHL and APA PsycINFO.

**Results:**

Key elements of effective mental health integrated care models in primary care are: Co-location of mental health and substance abuse services in the primary care setting, presence of licensed mental health clinicians, a case management approach to patient care, ongoing depression monitoring for up to 24 months and other miscellaneous elements. Key factors that contributed to the effective implementation of mental health integrated care in routine practice are the willingness to accept and promote system change, integrated physical and mental clinical records, the presence of a care manager, adequate staff training, a healthy organisational culture, regular supervision and support, a standardised workflow plan and care pathways that included clear role boundaries and the use of outcome measures. The need to develop sustainable funding mechanisms has also been emphasized.

**Conclusion:**

Integrated mental health care models typically have a co-located mental health clinician who works closely with the GP and the rest of the primary care team. Implementing mental health integrated care models in Australia requires a ‘whole of system’ change. Lessons learned from the Mental Health Nurse Incentive Program could form the foundation on which this model is implemented in Australia.

## Background

Mental disorders are among the most important public health problems in Australia. According to the Productivity Commission [1], over 23% of the population (5.9M) are at risk of developing a mental health problem. Persons with mental disorders also have a high rate of comorbidity, including psychological, social and physical problems [2]. In most developed countries, the first point of contact for individuals seeking healthcare is the general practitioner [GP].

More than 87% of the Australian population visit their GP every year [3]. The Royal Australian College of General Practitioners reports that 71% of the health problems discussed with GPs relate to psychological issues [4]. GPs also report that they are concerned about successfully managing patients’ mental health problems into the future [4]. Considering the burden of mental health problems encountered by GPs and the need to also manage co-morbidities in patients, an integrated approach to treatment is required. This approach needs collaboration between different health care specialists, health and community workers and agencies [5].

In the state of Victoria, Australia, mental health services have been reported to be fragmented and not fit for purpose thereby leaving a large group of individuals with an unmet need for services [6]. The COVID-19 pandemic exacerbated the poor state of mental health in the population when the state went into a 111-day lockdown [7]. The Royal Commission into Victoria's Mental Health System recommended that the system needed to be adaptive so that it ‘can identify and test new ideas, gather evidence about what works, and translate this into effective treatment, care and support’ [6]. The Commission envisaged that collaboration and communication occur between services within and beyond the mental health system and that continuing research, evaluation and innovation be used to respond to community needs [6]. As a response to the recommendations of the Royal Commission and the importance of general practitioners in primary mental health care, it is worth exploring mental health service models that involve general practitioners.

### Collaborative care [CC]

Over two decades ago, Michael Von Korff and David Goldberg argued that efforts to improve primary care of depression needed to focus on low cost case management dovetailed with accessible working relationships between the primary care doctor, the case manager and a mental health specialist [8]. The concept of multidisciplinary teams in primary care came to be referred to as a collaborative care model [CCM] and typically consisted of a multi-professional approach to patient care, a structured management plan, pharmacological and non-pharmacological interventions, scheduled patient follow-ups and enhanced inter-professional communication [9]. A systematic review in 2012 showed that CCMs could improve mental and physical outcomes for individuals with mental disorders and they provide a robust clinical and policy framework for care integration [10]. There have since been increased calls for this evidence to be translated into practice [11, 12].

The evidence for effective models of care for substance use disorders continues to be developed. For instance, the Washington State Hub and Spoke Model that incorporates primary care and substance use treatment programs, as well as outreach and referral has shown promise [13]. However, a systematic review of the effectiveness of models of care for the management of Alcohol Use Disorder in primary health care settings found that outcomes were mixed and that further research on the types and components of models was necessary [14].

Care coordination is a related recovery-oriented service delivery model that has been found to be effective in reducing unmet needs for persons with severe mental health problems [15]. It is built on the evidence that persons with severe and complex mental illness have multiple and complex needs related to accommodation, food, physical health problems, transport, childcare, etc. [16, 17]. It involves working with individuals to first identify and prioritize their needs, then liaising with multiple service providers to develop a care plan, and finally facilitating the provision of services according to that plan to meet clients’ needs [15]. This model considers the consumer as a whole including their culture, their family and carers as well as the community in which they live [18]. As a consumer-driven and co-designed model, it is meant to empower consumers and their carers in obtaining the best available care. National guidelines on coordinated care for persons with severe and complex mental illness have been published by the Australian government [18].

### Integrated care

Integrated care is different from CC. While CC refers to multiple professions working closely together in the delivery of care, in integrated care, the different professions are subsumed into a single organisational framework . Therefore, collaboration is a precondition for integration but collaboration does not require integration [19]. The literature on Integrated care for persons with mental illness is fast gaining traction [20]. According to a position paper by the American College of Physicians most integrated care models in the primary care setting fall into two major categories: the Collaborative Care Model (CCM), originally developed for the treatment of depression in primary care, and the Screening, Brief Intervention, Referral to Treatment (SBIRT) model for alcohol and substance use disorders (ASUD) [21]. Most other integrated models build on these two models. While the evidence on the effectiveness of the CCM has been demonstrated, effectiveness studies of the SBIRT have been less encouraging [22].

### General practice in Australia

In Australia, general practices have traditionally been smaller, private, single or two GP practices that provide comprehensive and continuing medical care. However, of late, they have started to become more corporatized [23]. The latest available data shows that there are 8147 general practices in Australia [24]. Nationally, the average number of full-time GPs is 117.7 per 100,000 population [25], the majority of whom work in group practices (82%) [26]. Currently, a majority (91%) of practices have practice nurses, 57% have allied health professionals and 23% have other specialists [26].

Females tend to see their GP more often than males, and older people visit their GP more regularly than younger people [26]. Patients speak highly of their GPs with 90% and over reporting that their GP always or often spends enough time with them and always or often shows respect, and listens carefully [26].

Primary health care and integrated care are a beneficial match. Valentijn et al. argue that by virtue of being continuous, comprehensive, and coordinated, primary health care is inherently integrated [27]. However, despite the known potential of these models of care, integrating mental (used interchangeably with behavioural) health care within the primary care setting has been fraught with challenges at the patient, provider and system level [28, 29]. Kates et al. argue that the different models themselves are not as important as the principles that underpin integrated care, and these principles can be translated to different settings [12].

This review focused on integrated care models that can feasibly be implemented in the Australian primary care setting, although the findings might have implications for other developed countries with similar primary care structures. The findings of this review can be used to support a consultative, evidence-based approach to commissioning, program design and implementation of primary mental health integrated care models.

## Method

A scoping review was undertaken since it is a method of evidence synthesis used to ‘map the literature on a particular topic or research area and provides an opportunity to identify key concepts, gaps in the research, and types and sources of evidence to inform practice, policymaking, and research’ [30]. The Joanna Briggs Institute [JBI] methodological framework for scoping reviews was adopted [31, 32]. JBI is an international organisation that promotes and supports evidence-based decisions to improve health and health service delivery.

### Aims of the review

The aims of this review were twofold:

1. To describe the models of integrated care including care coordination within a primary care setting that have demonstrated evidence of a positive effect on improving: mental health and wellbeing, substance abuse.
2. To identify factors contributing to the effective implementation of integrated care models within primary care that are relevant to the Australian setting.

### Inclusion criteria

The inclusion criteria have been categorised under the three headings of participants, concept and context as per the JBI guidelines [31, 32].

#### Participants

Participants in included studies were persons with mental health problems, typically common mental disorders such as depression and anxiety. No restrictions were placed on age or gender of participants since the focus was to identify effective models of integrated care in the primary care setting.

#### Concept

The main concept of this scoping review relates to models of integrated mental health care including care coordination models that have shown benefit in improving mental health and wellbeing and substance abuse. With a view to trialling such models, commissioning agencies are also interested in identifying the factors contributing to their effective implementation such as funding and infrastructure, service access and intake, workforce, technology, research, evaluation and performance monitoring, lived experience engagement, governance; and partnerships.

#### Context

The context of this review includes primary care settings in developed countries that are comparable to the Australian setting. Dates for the search were customised from 1st January 2000 to 7th June 2023 since some of the early publications of collaborative care were after year 2000 [8]. Language was restricted to English for convenience. Inclusion and exclusion criteria are given in Table 1.

**Table 1:** Inclusion and exclusion criteria

Inclusion criteria	Exclusion criteria
Primary Health Care	Autism
Family Practice	Dementia
Primary Care Nursing	Alzheimer's
Integrated care	Foetal health
Integrated care models	HIV/AIDS
Patient-Centered Care	Cancer
Care coordination	Hepatitis
Mental Disorders	Motivational interviewing
Mental Health Services	Gambling disorder
Substance-Related Disorders	Domestic violence
Children	Trauma care
Adults	Contraception
Elderly	Low- and Middle-Income Countries
Developed countries	Cognitive Impairment
	Prisons and Justice system
	Suicide as the primary focus
	Veteran’s Affairs

### Search strategy

The search strategy was organised around the three concepts of Primary health care, Integrated care models/care coordination and Mental disorders. MeSH terms and keywords were chosen and piloted in PubMed. Articles identified through this process were assessed to further refine the search strategy. Search terms and keywords within each concept were combined using the Boolean operator ‘OR’ and across components using ‘AND’. The master search was designed and finalised in PubMed. The searches were then converted and executed in CINAHL and APA PsycINFO databases as described in Box 1.

### Study selection

All references were imported to Covidence for screening and study selection. Duplicates were then removed.

#### Title and abstract screening

Following discussions on screening protocols, title and abstract screening was performed independently by two reviewers [AI and EM] and conflicts were resolved following discussion.

#### Full-text screening

Full text screening was also undertaken independently by two reviewers. The lead author reviewed the excluded papers a second time. We excluded reports from Veteran’s Affairs settings in the USA because they are typically large clinics with multiple professionals working under a single roof, whereas Australia has smaller practices with one or more GPs assisted by one or two nurses and administrative staff.

#### Data extraction

Reports were forwarded for data extraction following full text reviews. Only those that reported on effective mental health integrated care models in primary care settings or factors that contributed to their effectiveness were included.

Box 1: Search terms for databases ***PubMed search***

(("Primary Health Care"[Mesh] OR "Family Practice"[Mesh] OR "Primary Care Nursing"[Mesh]) AND ("Delivery of Health Care, Integrated"[Mesh] OR "Models, Organizational*" OR "Patient-Centered Care"[Mesh] OR "Care coordination"[tw])) AND ("Mental Disorders"[Mesh] OR "Mental Health Services"[Mesh] OR "Substance-Related Disorders"[Mesh])

Filters: Clinical Trial, Meta-Analysis, Randomized Controlled Trial, Review, Systematic Review, Humans, English

***CINAHL complete search***

(“Primary health care” OR “primary care”) AND “Integrated care models” AND “Mental Disorders”

***PsycInfo search***

(("Primary Health Care" or "Family Practice" or "Primary Care Nursing") and ("Integrated care" or "Integrated care models" or "Patient-Centered Care" or "Care coordination") and ("Mental Disorders" or "Mental Health Services" or "Substance-Related Disorders")).mp. [mp = title, abstract, heading word, table of contents, key concepts, original title, tests & measures, mesh word]

#### Data collation and synthesis

Studies identified in the review were divided into two groups. Experimental studies (RCTs) and systematic reviews of experimental studies that reported on effective mental health integrated care models in primary care settings were included in Group 1. Group 2 included reviews of characteristics of organisations that have achieved mental health integrated care mechanisms in primary care, facilitators and barriers for implementing this integration as well as recent advances on how to establish such models in routine care. Findings from group 1 and group 2 studies were then extracted separately into tables and synthesised as follows.

1. Elements of mental health integrated care models in primary care settings that have shown improvement in patient outcomes
2. Key factors that contribute to the effective implementation of such models that are relevant to the Australian primary care setting.

## Results

**Fig. 1:**
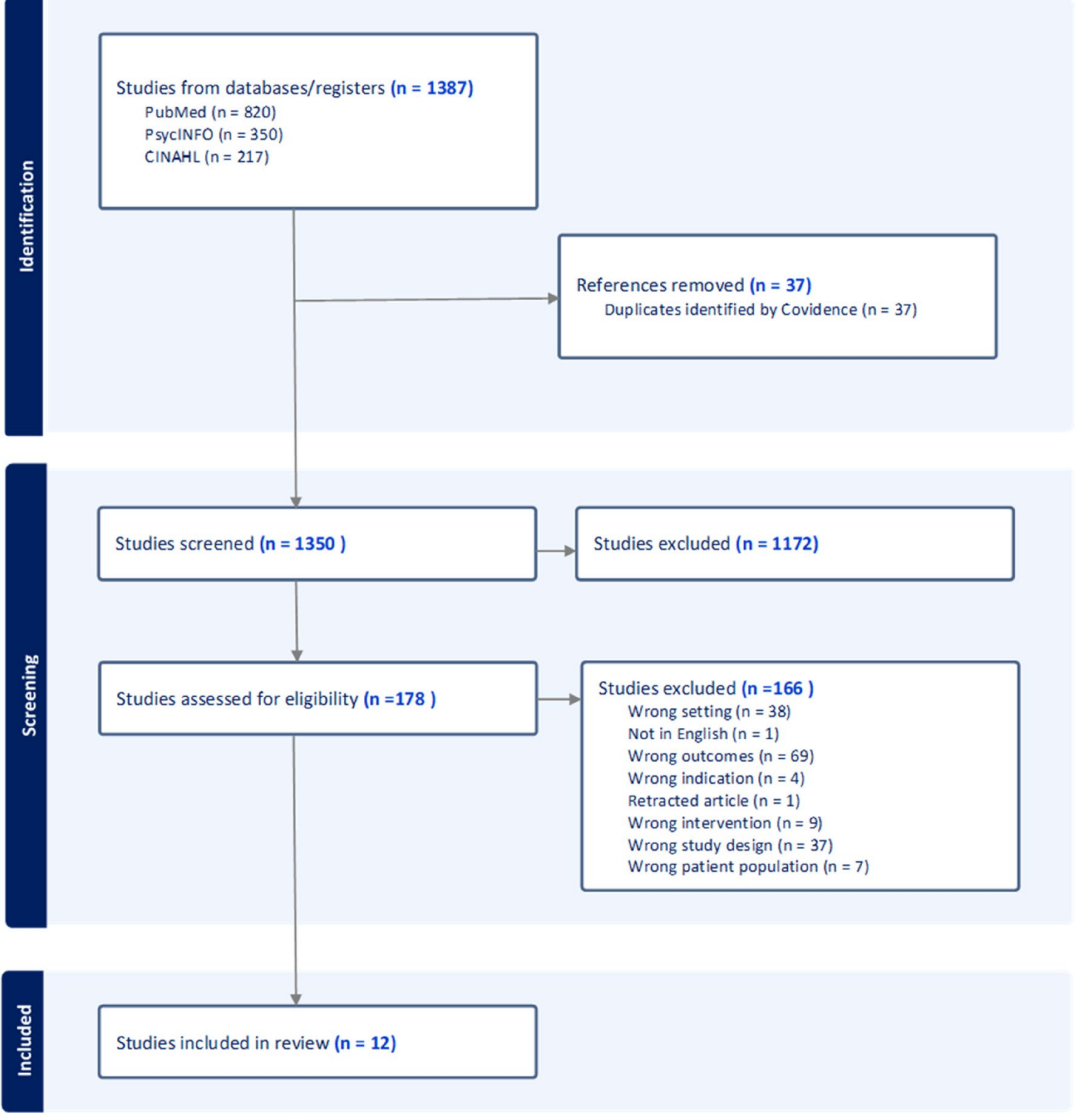
PRISMA chart [Covidence] showing selection of studies for the scoping review

One thousand three hundred and eighty-seven studies were identified in the search. Twelve papers were selected for the review. They included 2 randomised controlled trials, 2 cluster randomised controlled trails, one systematic review of randomised controlled trials, 2 systematic reviews, one systematic review of interviews with service providers, 2 integrative reviews, one narrative review and one site specific analysis. Six studies were based in the USA, 2 in the UK and one each in Australia, Netherlands, Germany and China. The report from China was included because it reviewed models from around the world rather than from low- and middle-income countries. Figure 1 shows the PRISMA flowchart for selection of reports for this review.

### Elements of mental health integrated care models in primary care (Group 1)

Six studies described mental health integrated care models in primary care settings that showed improvement in patient outcomes [33–38]. Characteristics of each model studied and their reported outcomes are given in Table 2. Outcomes reported by these studies primarily include better engagement of patients with mental health and substance abuse services [33, 34] and better clinical and functional outcomes [35, 36]. Key elements of effective mental health integrated care models in primary care are given below.

**Table 2:** Elements of effective mental health integrated care models in primary care settings and their outcomes

Reference, Country Study type	Model/study name	Aim and sample characteristics	Elements of the model	Reported outcomes
Bartels, Coakley [34], USA Randomized Controlled Trial Ayalon, Arean [33], USA Site specific analysis	Primary Care Research in Substance Abuse and Mental Health for the Elderly (PRISM-E) study	Bartels et al., (2004) aimed to test whether relative to the control, the integrated model of care would result in better access to and utilization of Mental health /substance use services in both black and white older adults (> 65 years) Ayalon et al., (2007) examined data from 1 of the sites from this trial to compare engagement and participation rates in black and white elderly	Co-location Licensed mental health clinicians Communication between providers Timely cross appointments	Integrated care was associated with more mental health and substance abuse visits per patient Persons with more severe depression and more severe problem drinking showed greater engagement Patients were more likely to have first appointments with mental health and substance abuse care provider within 2 weeks of the primary care visit Integrating mental health/substance abuse services were particularly effective in engaging black elderly in mental health/substance abuse services
Areán, Ayalon [35], USA Randomised Controlled Trial	Improving Mood-Promoting Access to Collaborative Treatment (IMPACT) study	To test whether depressed older adults from the 2 largest ethnic minority groups (black and Latino) benefited similarly from CC for depression in primary care as did whites	Patient education Co-located mental health professional Ongoing depression monitoring Regular specialist consults	By the 12-month assessment period, older minorities who received CC used far more guideline concordant depression services (antidepressant medications or psychotherapy) Older minorities who received CC had significantly better depression outcomes, significantly higher rates of treatment response, and significantly higher rates of remission than minorities in usual care (UC) Blacks who received CC had substantially better functional outcomes than did blacks in UC Provision of services in a nonmental health setting, such as primary care medicine addresses stigma and trust barriers Older minorities tend to be wary of the mental health system and are more likely to seek services from their primary care physician
Gensichen, von Korff [36], Germany, Cluster randomised controlled trial	PRimary care Monitoring for depressive Patients Trial (PRoMPT)	To test whether case management by a practice-based health care assistant (HCA) can reduce depression symptoms and improve the process of care for adult patients with major depression in small primary care practices. HCAs have less training than U.S. physician assistants or nurse practitioners. In Germany, HCAs have 3 years of on-the-job training. They are mainly responsible for administrative tasks in general practice but provide basic clinical procedures	Training Case management Encouragement for self-management Communication with family physician	This is a simple, depression case management intervention in non-academic, non-structured, small primary care practice settings Patients who received telephone case management by HCAs reported slightly greater improvements in depression symptoms, better adherence to antidepressant therapies, and more favourable assessments of the quality of their care than did patients randomly assigned to receive usual care The practice teams’ familiarity with their patients and long-time continuity of the patient–provider relationships, which are typical for small primary care practices, may have played a role in achieving the positive results
Huijbregts, de Jong [37], Netherlands, Cluster randomized controlled trial	Effectiveness of the IMPACT model in primary care in the Netherlands	To test whether the IMPACT collaborative care model developed in the USA could be applied for adults (> 17 years) in small, individual practices such as those found in the Netherlands	Multidisciplinary team Standard treatment intervention Target driven treatment Use of a web-based decision aid with stepwise algorithm Consultation by a psychiatrist	CC care was more effective in achieving treatment response than CAU at three months for the total group of patients who received collaborative care. The effect was not statistically significant at 6 and 12 months The effect of CC, particularly for the screened group, subsided to a certain extent after twelve months, which might be explained by the fact that the intensity of the intervention was toned down at this point The treatment response and remission in the usual care group was very low (the response percentages ranged between 10.5% and 25.8%). ‘Depression’ may not have been on the patients’ agenda in their contacts with primary care in the CAU-condition, as these cases were detected by means of screening
Chang-Quan, Bi-Rong [38], China, Systematic review of randomized controlled trials	Collaborative Care Interventions (CCI) for Depression in the Elderly	To determine the effective components and the feasibility of CCIs in the treatment of depression in older patients Participants–Pool A studies 1801 primary care patients aged 60 and older with major depression, dysthymia, or both from 18 primary care clinics in 8 US health care organizations Excluded criteria: current drinking, problems, bipolar disorder or psychosis, severe cognitive impairment, acute risk of suicide, or ongoing psychiatric treatment Participants—Pool B studies 598 primary care patients aged 60 and older with depression from 20 primary care practices in New York City, Philadelphia, and Pittsburgh regions Excluded criteria: current drinking, problems, bipolar disorder or psychosis, severe cognitive impairment, acute risk of suicide, or ongoing psychiatric treatment	Interventions—Pool A studies Collaborative Care Intervention (IMPACT) Intervention—Pool B studies Prevention of Suicide in Primary Care Elderly (PROSPECT)	CCIs significantly decrease suicide ideation compared to those receiving usual care CCIs significantly increased depression-free days, but did not significantly increase outpatient cost Collaborative care interventions with communication between primary care providers and mental health providers were no more effective in improving depression symptoms than CCIs without such communication

Co-location: Mental health and substance abuse services were co-located in the primary care setting (including assessment, care planning, counselling, case management, psychotherapy, and pharmacological treatment), with no distinction in terms of signage or clinic names [33–35].

Licensed mental health clinicians: Mental health and substance abuse services were provided by licensed mental health/substance abuse providers (including social workers, psychologists, psychiatric nurses, psychiatrists, and master’s-level counsellors) [33, 34] although in one study, trained health care assistants (HCA) provided case management [36]. In addition to having a licensed mental health practitioner on site, some models had visiting or liaison psychiatrists particularly when patients presented with complex conditions [35, 37, 38].

Case management: A case management approach where proactive support was provided for the patient by the entire practice team was evident. This required communication between mental health and substance abuse clinician and primary care provider about the clinical evaluation and treatment plan [33, 34] or between the case manager and general practitioner [36]. It also required regular cross appointments [33, 34].

Ongoing depression monitoring: Patients were typically monitored every 2 weeks during the acute phase of treatment and then monthly for 1 year after stabilization of depression as per the Agency for Health Care Policy and Research guidelines for the treatment of depression in primary care [35]. In the study by Gensichen, and colleagues [36] HCAs contacted their patients by telephone twice a week in the first month and then once a month for the following 11 months. They monitored depression symptoms and adherence to medication by using the Depression Monitoring List. In other studies, patients received proactive depression treatment in primary care for 12 months and were followed up for 12 or 24 months [38].

Miscellaneous: There were other elements that were part of individual studies such as patient education in the form of video and written information [35], encouragement to take part in self-management activities, such as medication adherence and pleasant or social activities [36], adopting a standard treatment intervention such as the Problem Solving Treatment [37] or the use of a web-based patient tracking system with stepwise algorithm to monitor medication adherence and patient progress [37].

### Factors that contributed to the effective implementation of mental health integrated care in primary care settings (Group 2)

Six reports explored factors that contributed to the effectiveness of mental health integrated care models in primary care settings [22, 39–43]. Characteristics and findings of each study are given in Table 3. Key factors that contributed to the effective implementation of mental health integrated care are described below.

**Table 3:** Characteristics and findings of studies that explored factors that contributed to the effective implementation of mental health integrated care models in primary care settings

Reference, country,	Type of study	Aim of the study	Findings
Martin, White [39]	Systematic review	To review how the following components of integration are implemented and practiced a) collaboration practices, b) program models, c) interventions, d) provider type, e) training and supervision practices, and f) setting	Collaboration practices: are implemented in the form of communication between primary care providers [PCP] and behavioural health providers [BHP], or PCPs providing referrals to onsite BHPs or referral to BHPs as a “warm handoff.” BHPs also offered treatment recommendations to PCPs or psychiatrist consultations were made available to PCPs through a shared-decision making process Program Models: Use of treatment guidelines or a pre-existing model such as the PRISM-E or IMPACT Behavioural Health Interventions: include psychoeducation, psychotropic medication, care management strategies, follow-up contact with BHPs with patients after treatment, some type of psychotherapy (e.g., behavioural, cognitive– behavioural, brief, group) and family therapy used alone or in combination with others Behavioural Health Training and Supervision: Some type of behavioural health training or supervision was common. Those who were trained included BHPs, and PCPs to deliver mental health treatment, some type of supervision for model fidelity, a psychiatrist or psychologist supervisor, weekly supervision, team-based supervision, or the use of a treatment manual Behavioural Health Provider Types: included nurses, psychiatrists, psychologists, and social workers. Nurses either worked alone or together with social workers or psychologists Setting: Most studies reported a primary care setting but other settings included Veteran’s Affairs Medical Centre, rural communities, suburban communities, urban communities, community health centres and outpatient patient hospital networks
Grazier, Smiley [40] USA	Systematic review	To identify characteristics of organizations that have successfully integrated mental health and primary care	Prioritized underserved and vulnerable populations Used data-driven best practices Community-Wide Collaboration Support from Influential Leaders and Established Institutions Team Approach That Includes the Patient and Family Diverse Funding Streams
Wood, Ohlsen [41] UK	Systematic review	To determine patient, staff or organisational factors that act as barriers/facilitators o the implementation of Collaborative Care for patients with depression in primary care	Barriers Organisations’ readiness for change Lack of understanding of Collaborative Care Patients finding it challenging to engage with screening tools and self-help material Breakdowns in networks and communication pathways Facilitators Positive staff attitudes to change particularly when one of the senior physicians took the role of championing the service to his/her colleagues Clearly developed and defined role of case manager seen as efficient and effective Key characteristics needed within the role of the case manager Having structured management plans for patients Staff involved have sufficient training on the intervention and what can be expected from it Finding the right screening and outcome tools and training all staff in how and why these tools were being used Having a standardised care pathway; GPs were more likely to be happy to talk about depression if they knew what to do once it was identified Case managers and staff reported confidence in the specific interventions available and being able to see their benefits Co-location improved communication and helped de-stigmatise mental health treatment Integrated information systems also helped as it made it easier to share notes and pass messages to colleagues A supportive, constructive and regular supervision schedule helped the case managers deliver care and talk over difficult cases or ask questions about referral on to mental health services where required Scheduled follow ups and someone taking responsibility to ensure that happened was beneficial The CC intervention had to be seen either as revenue neutral or revenue enhancing for organisation's financial buy in
Ramanuj, Ferenchik [22] UK	Narrative review	Recent mechanisms to incentivize integration efforts between health and social services in primary care	Novel approaches to integrated care are uncommon Most current models have been facilitated by research funding, pump primed grants, or other time-limited financial levers Few valid and feasible process and outcomes measures exist to support integrated care Current quality-outcome measures that do exist tend to focus on single-disease entities or populations, rather than reflecting the reality of multimorbidity in this population The need for payment reform and the development of quality metrics need to be addressed Telemedicine, along with incentives to recruit and retain providers, could be a means to address workforce challenges New health information technology (IT) strategies must also be employed to support continuous and coordinated care and monitoring Integrated electronic health records (EHR), are needed to integrate medical and behavioural health information streams with telehealth, social services, prisons, and schools, for example Successful implementation of integrated care must be supported at multiple levels—policy, practice and provider and are usually built around shared goals and formal partnerships between providers
Coates, Coppleson [42] Australia	Integrative review	To identify factors that support the implementation of integrated care between physical and mental health services in real-world settings	Adequate resourcing: Care coordinators (particularly in rural areas) were challenged by very busy schedules, with many responsibilities and limited time. An integrated model with shared responsibility, where psychiatric nurses were stationed in a residential home but employed by specialist mental health services, was more effective than a model where the residential home employed their own psychiatric nurses Shared values: Implementation efforts were impeded by differences in opinion and conflict around who controls patient provider relationships, the structure of the care provision, how care notes should be completed, and how patient perspectives should be managed. These differences affected implementation and resulted in high rates of staff turnover Effective communication between staff: Communication between staff was reported as a key barrier to successful implementation particularly when they have different professional backgrounds. Effective communication requires formal and informal opportunities to exchange information so that organizational, clinician, and patient objectives can be met Regular formal meetings between leaders and frontline staff: and responsive e-mail communication helped to address and resolve conflicts as they arose IT infrastructure: The ability to share electronic medical records, is critical, for successful integration and can facilitate communication between staff. However, the roll out of integrated IT systems can take time, is complicated, and costly Flexible administrative organizations: For integrated care to be effective, organizations and employees need to be flexible and to step outside of prescribed roles to work together in creative ways. Integration efforts can be impeded by reluctant administrators who are unable or unwilling to be flexible Role clarity and accountability: Complex integrated care models with multi-agency leadership and accountability can lead to concerns over lines of accountability and role confusion for staff, hindering implementation, staff engagement and training. Integrated care coordinators have expressed a lack of understanding of their role and were confused about their responsibilities Staff engagement and training: When staff are not consulted or involved in the planning of the model, or lack confidence or skills required to implement the model, they can become disengaged. Limited knowledge of allied health providers on physical health and limited knowledge of primary care providers on mental health has shown to impede integration. These skills and knowledge gaps can be addressed with training
Peer and Koren [43] USA	Integrative review	To summarize and critically examine factors that impact the integration of mental health care into primary care settings, to advance evidence-based knowledge, and promote awareness to ensure the successful implementation of such integrative models	Patient-centered care: A focus on the patient’s needs through the use of a tailored care plan is crucial in caring for a complex population with different needs and is noted as an imperative facilitator Targeting vulnerable populations, such as ethnic minorities, children and adolescents, or people with medical comorbidities, who may face unusually great barriers regarding access to care, is important Staff and providers’ attitudes towards the patient’s culture, including any stigma regarding mental health and substance use disorders also affects the success of the program Relationships: among the clinicians themselves is vital to promote collaboration between the disciplines, such as interdisciplinary huddles and ‘warm hand-offs’. A second was the relationship between the leadership and the clinicians and staff. Without ongoing support from the leadership and stakeholders, integration will not succeed, and motivation for its success will decrease among staff. A third relationship was between the clinicians and the patients, where the lack of a good relationship that fosters trust was found to pose a barrier to effective treatment. Finally, the lack of a strong relationship between the clinic and the community, in the form of collaborations, presented another barrier to successful integration Physical accessibility: The lack of designated and welcoming spaces for behavioural health care was found to be a barrier to engaging patients. Behavioural health services that were not co-located with primary care, and that were sometimes in a different and remote building posed another challenge to service availability and close collaboration between the primary and the behavioural care teams. Remote access to public transportation and lack of parking spaces were also noted as travel-related and accessibility barriers Operation and infrastructure: Flexible scheduling and availability of mental health clinicians in the form of late appointments and walk-ins are vital in the treatment of patients with mental illness or a behavioural crisis. Flexible scheduling that allows for treating all patients with sufficient visit time, regardless of the visit type, is a key element for the successful integration of behavioural treatment into primary care A standardized workflow plan: that monitors outreach, progress, and outcomes for each patient, in addition to the performance of standardized mental health screening; all of these can be incorporated into the EMR as tracking tools. Clinics that developed a protocol for mental health screening and followed a standardized work-flow plan, including a plan for transitioning between different care groups, managed to better engage in the care of mentally ill patients Electronic medical records: Underperforming templates and insufficient technology support, has been reported as a major barrier to a successful implementation of the integrated model in many studies. An inadequately integrated EMR that fails to allow access to all clinicians and staff was noted as an additional barrier. Both the primary and the behavioural care staff reported that workarounds in a non-integrated EMR resulted in wasted staff time, particularly when accessing multiple data resources. Technology support could provide appropriate protection for sensitive data Training: Adequate staff training, specifically in behavioural health, is crucial for caring for patients with mental illnesses. Ill-prepared staff, staff that lacked motivation, and new graduates that had not been prepared or trained to treat the complexity of these illnesses were among the challenges encountered in training the staff. Cross-training between the primary care and the behavioural staff was found to be beneficial in preparing the staff and increasing empathy towards patients Team approach: Collaboration between the primary care and the mental health clinicians and staff, such as an interdisciplinary team and shared care plans, is an important advantage that integrated care has over two separate care teams. The advantage of a larger care team that included case management, patients, and the families of patients allowed for a more comprehensive and holistic view of the patient’s needs Staffing: A diminished workforce, specifically, staff trained in behavioural health, can result in staff burnout and high turnover. Psychological relief through group cohesion and working on personal growth through education has benefits. Issues of retaining the staff also stemmed from unclear staff roles. The resulting confusion occurred mainly between primary care staff and the behavioural team staff. Primary care providers have reported uncertainty about their responsibilities regarding diagnosing and treating mental illnesses. One of the solutions offered was the assistance of an integration champion, which can further facilitate integration and can address any confusion, thus benefiting the organization Funds and health insurance: Insufficient reimbursement is a key barrier to a successful integration; either in the form of low Medicaid/ Medicare rates, fee-for-service, or insufficient coverage by private insurance. For example, clinicians have reported being reimbursed the same for behavioural services as for primary care, although behavioural services require longer visits and staff time. Because of the lack of equitable billing regulations and reimbursement for behavioural care, many clinics could not sustain integrated care. In addition, limited sources of funds and insufficient funds were barriers to sustainability and for retaining clinicians and staff especially in small clinics

#### Willingness to accept and promote system change

General practices need to be willing to accept and promote system change [41, 44]. It is important for GPs to recognize the benefits of the model [36, 41] and have shared values with staff [42]. This is possible when there are individuals with deep institutional vision such as influential supporters of general practices (e.g. political and other leaders) [40]. Such individuals can serve as champions to encourage GPs to refer their patients with depression to the care manager [41, 45] or facilitate integration and address any confusion, thus benefiting the organization [40, 43, 46]. Higher-level agencies can also provide practices with strong support for system change [40].

#### Presence of a care manager

Having a care manager on site and who is easily accessible was significantly correlated with activating patients into the program [41, 45].

#### Adequate training

Adequate staff training, specifically in mental health, is crucial for caring for patients with mental illnesses [42, 43]. Cross-training between the primary care and the behavioural staff was found to be beneficial in preparing staff and increasing empathy towards patients [43, 46].

#### Standardised workflow plan and care pathways

Clinics that developed a protocol for mental health screening and followed a standardized work flow plan, including a plan for transitioning between different care groups, managed to better engage in the care of mentally ill patients [22, 41, 43, 47]. In addition, clear role boundaries where the mental health clinician was not perceived as taking over the patient's depression care (e.g. GP decided when to change the dosage or type of medication) [41, 48], scheduled follow-up appointments for continuing engagement [41, 44] and the use of outcome measures such as the Patient Health Questionnaire-9 were found to enable the implementation of mental health integrated care. The PHQ-9 is a validated and widely used screening tool for depression in primary care [49]. It is the most accepted outcome measure by GPs, which gives them confidence in their ability to provide high quality depression care [41, 50].

#### Consolidated physical and mental clinical records

Integrated electronic health records showed potential to expand patient care beyond the traditional primary care setting by integrating medical and mental health information streams with telehealth, social services, etc. [22].

The ability to share electronic medical records was also found to contribute to effectiveness of the model [41, 42, 47].

#### Healthy organisational culture

A good relationship between the medical and the mental health care providers is vital to promote collaboration between the disciplines. This often took the form of ‘interdisciplinary huddles’ or daily catch-ups to discuss new patients and issues from the previous day [43]. Effective communication between service providers which was either written, verbal, formal or informal contributed to the healthy environment and smooth running of the model of care [42]. Similarly, organisational culture was enhanced when staff had the ability to step outside of their prescribed roles and work creatively [42] but at the same time had role clarity and accountability [42].

#### Regular supervision and support

Regular and ongoing supervision and support is considered to maintain morale and motivation [41, 51]. Support and supervision could be in the form of regular site visits and addressing implementation challenges [41, 52]. However, this required a good relationship between the leadership and the clinicians and staff [43].

## Discussion

This review sought to first describe the models of integrated care within a primary care setting that have demonstrated evidence of a positive effect on improving mental health and wellbeing, and substance abuse challenges and second to identify the factors contributing to their effective implementation. Integrated care models typically have a co-located mental health clinician who works closely with the GP and the rest of the primary care team. Factors that enabled these models to be translated to routine care included, a willingness to accept and promote system change, consolidated physical and mental health records and regular supervision and support.

A scoping review similar to ours was published after our review was completed. The findings of which were broadly similar to ours and included themes related to funding, health practitioner workforce/training, and relationships with initiatives, organizations, and communities [53].

A key finding of this review was that despite integrated care models showing clear evidence of effectiveness in research settings, there continues to be challenges in translating them into routine practice [22, 54]. The services that have succeeded in doing so had the rather unique characteristics of a positive organisational culture and vision [40]. In Australia, integrating mental health care into primary care could work better for larger practices where a business case for this model might prevail. Primary Health Networks, which are agencies that aim to strengthen the primary health care system in Australia, are best placed to lead this change [55].

In countries such as the UK and Australia, the CC model was translated as the Mental Health Nurse Incentive Program [MHNIP], where mental health nurses were co-located at general practices to facilitate access to mental health services [56–59].

The model had several promising outcomes such as symptom reduction, improved coping and relationships, and enhanced community participation [56, 60]. However, there were challenges with the funding models [61] and in some countries, nurse burnout [62] and retention issues [63]. Nonetheless, this type of model resulted in improvements in integration, clinical effectiveness, patient-centred care, access and efficiency [63]. Mental health nurses continue to be excited about working in primary care, have more autonomy and flexibility in their role and have more time for health promotion [64].

A case has been made for new payment models to be developed that enable implementation of mental health integrated care because effectiveness of these models has thus far been demonstrated mostly with research funding [22]. In Australia, the mainstream mental health system is ‘block funded’ by state governments while primary care is mostly paid for by the Australian Government through fees for service, and medication subsidies [56]. Bringing mental health care into the primary health setting will therefore require a new funding model.

There are several barriers to seeking help for problems related to substance abuse [65]. These include the lack of awareness of the problem, inadequate social support, fear of treatment, privacy concerns, and lack of treatment availability [65]. In addition to these barriers, stigma attached to substance abuse problems and seeking help for it is experienced more intensely by disadvantaged communities [66]. By encapsulating mental health care within the primary care service, integrated care models overcome these barriers [40].

There are a few instances where organisations have included peer workers in their integrated health care service models [67], although how and where they fit into the care model is not yet clear and their effectiveness in improving mental health outcomes in the primary care setting is yet to be proven [68]. Patient-Centered Medical Homes (PCMH) are an improved version of general practices that focus on patient needs rather than just their medical issues [69]. However, this model is a work in progress and has yet to demonstrate effectiveness in managing persons with mental health challenges [70]. Stepped care has been trialled in integrated care settings and has shown to be cost effective [71]. However, this intervention is also in the early stages of development.

## Conclusions

Integrated mental health care models typically have a co-located mental health clinician who works closely with the GP and the rest of the primary care team. Implementing mental health integrated care models in Australia requires a ‘whole of system’ change. Lessons learned from the MHNIP could form the foundation on which this model is implemented in Australia.

## Data Availability

The datasets used and/or analysed during the current study are available from the corresponding author on reasonable request.
